# Recurrent oropharyngeal squamous cell carcinomas maintain anti-tumor immunity and multinucleation levels following completion of radiation

**DOI:** 10.21203/rs.3.rs-3267009/v1

**Published:** 2023-08-21

**Authors:** Patricia Castro, Germán Corredor, Can Koyuncu, Luke A. Nordstrom, Michelle Tiji, Taylor Leavitt, James S. Lewis, Anant Madabhushi, Mitchell J. Frederick, Vlad C. Sandulache

**Affiliations:** Baylor College of Medicine; Georgia Institute of Technology and Emory University; Georgia Institute of Technology and Emory University; Operative Care Line, Michael E. DeBakey Veterans Affairs Medical Center; Operative Care Line, Michael E. DeBakey Veterans Affairs Medical Center; Baylor College of Medicine; Vanderbilt University Medical Center; Georgia Institute of Technology and Emory University; Baylor College of Medicine; Operative Care Line, Michael E. DeBakey Veterans Affairs Medical Center

**Keywords:** oropharyngeal cancer, multinucleation, tumor infiltrating lymphocytes, recurrence

## Abstract

**Objective:**

Oropharyngeal squamous cell carcinoma (OPSCC) recurrence is almost universally fatal. Development of effective therapeutic options requires an improved understanding of recurrent OPSCC biology.

**Methods:**

We analyzed paired primary-recurrent OPSCC from Veterans treated at the Michael E. DeBakey Veterans Affairs Medical Center between 2000 and 2020 who received curative intent radiation-based treatment (with or without chemotherapy). Patient tumors were analyzed using standard immunohistochemistry and automated imaging of infiltrating lymphocytes and multinucleated tumor cells coupled to machine learning algorithms.

**Results:**

Primary and recurrent tumors demonstrated high concordance via p16 and p53 immunohistochemistry, with comparable levels of multinucleation. In contrast, recurrent tumors demonstrated significantly higher levels of CD8+ tumor infiltrating lymphocytes (p<0.05) and higher levels of PD-L1 expression (p<0.05).

**Conclusion:**

Exposure to chemo-radiation and recurrence following treatment does not appear deleterious to underlying biological characteristics and anti-tumor immunity of oropharyngeal cancer, suggesting that novel treatment regimens may be as effective in the salvage setting as in the definitive intent setting.

## Introduction

Infection with high-risk subtypes of human papillomavirus (HPV) has driven an epidemic rise in the incidence of oropharyngeal squamous cell carcinoma (OPSCC),^1–5^ a disease formerly associated exclusively with heavy tobacco exposure.^6,7^ Although a majority of new HPV-associated OPSCC diagnoses occur in patients with either no history of tobacco exposure or limited past exposure, select patient populations including Veterans and indigent patients maintain high rates of tobacco exposure and concomitantly high rates of tobacco associated cancers.^7–11^ In the context of OPSCC, tobacco exposure that exceeds 10 pack years cumulatively prior to diagnosis generates an “intermediate-risk” phenotype that is associated with a nearly 25% reduction in survival across both prospective and retrospective cohorts.^12–15^ This phenotype, exacerbated by extensive tobacco exposure exceeding 30 pack years, has been shown by us to drastically reduce survival in Veteran cohorts despite equivalent deployment of combined multi-modality treatment (chemo-radiation).^7–8^

We previously robustly linked survival and chemo-radiation response in OPSCC to a combination of intrinsic tumor biological factors, namely the presence of multinucleated cells and geo-spatial organization of infiltrating lymphocytes.^8,16–19^ Although these factors have been linked by our group with survival in low-risk and intermediate-risk HPV-associated and high-risk HPV-independent OPSCC, it remains unclear whether these biological features are impacted by completion of curative intent treatment or whether they are retained post-treatment.

Intermediate- and high-risk OPSCC demonstrate high rates of in-field or locoregional recurrence consistent with other HPV-independent disease sites such as oral cavity.^7,20^ The viability of salvage treatment whether it consists of surgery, re-irradiation, or systemic treatment with chemotherapy with or without immune checkpoint inhibitors is driven by relative therapeutic index (anti-tumor effectiveness / excess toxicity). Anti-tumor effectiveness of OPSCC treatment has now been linked by us and others to intrinsic tumor biology and anti-tumor immunity.^7,8,16–18,21^ In the current manuscript we sought to determine whether definitive, curative intent chemo-radiation generated a deleterious shift in tumor biology and immunity in the context of OPSCC enriched for intermediate- and high-risk disease that could negatively impact the effectiveness of salvage treatment.

## Materials and Methods

### Clinical dataset.

Following approval from Baylor College of Medicine and the Michael E. DeBakey Veteran’s Administration (MEDVAMC) Institutional Review Boards, we reviewed the records of Veterans with previously untreated oropharyngeal squamous cell carcinoma (OPSCC) between January 1, 2000 and January 1, 2020. Data collection and analysis was performed in a manner consistent with existing standards for clinical research (Declaration of Helsinki, US Federal Policy for the Protection of Human Subjects). Inclusion criteria included: 1) primary OPSCC, 2) tissue diagnosis at the MEDVAMC, 3) treatment delivery at the MEDVAMC, 4) at least 2 years of post-treatment surveillance at the MEDVAMC and 5) documented recurrence/metastasis at the MEDVAMC. All patients were required to have tissue available for diagnostic assessment and performance of research studies. Since the research testing required tumor tissue with intact morphology, patients that underwent diagnosis of primary tumor or recurrent disease via fine needle aspiration were excluded.

Due to the retrospective nature of the study and the minimal risk profile, waiver of consent was obtained from the IRB prior to research study commencement. Exclusion criteria included treatment at an outside institution and lack of recurrent disease. Demographic information including age, gender, race, smoking history and alcohol consumption was collected and analyzed. Clinical and pathologic features were collected using the American Joint Commission on Cancer (Staging Manual 7th Edition) staging system. Given the high preponderance of high- and intermediate-risk OPSCC, the 7th Edition provides a more nuanced and clinically relevant staging summary of OPSCC compared to the newer 8th Edition, which drastically de-escalated N-classification regardless of relative tumor risk.^22^ Results of diagnostic results and biopsies, treatments delivered and the associated dates were recorded.

### Quantitative immunohistochemistry.

We have previously described our quantitative assessment of p16 immunohistochemistry along with quantitative measurements of infiltrative CD3 + and CD8 + lymphocytes.^19^ Briefly, formalin-fixed paraffin-embedded (FFPE) biopsy tissue blocks were retrieved from the archive maintained at the MEDVAMC Department of Pathology. Slides were deparaffinized and rehydrated prior to the blocking of endogenous peroxide activity. Immuno-histochemical (IHC) stains were performed on an automated tissue-staining system using the Bond Polymer Refine Detection (Leica Biosystems, Buffalo Grove, IL) using antibodies for CD3: LN10, CD8: 4B11, p53: DO-7, PDL1: 73 – 10 (Leica Biosystems, Richmond IL), and p16 E6H4 (Ventana, Tucson, Arizona). Representative images were captured using the Vectra3 scanner using the CRI multispectral camera using a 20x UplanSApo objective (0.75 aperture) mounted on an Olympus microscope. (Akoya Biosciences, Menlo Park, CA) and analyzed using InForm 2.4 software (Akoya). The percent of positively stained features was calculated as the number of positive cells divided by the total number of cells in the tumor segmented areas. p53 positive cells were characterized by strong nuclear staining by IHC in the tumor areas of the tissue. A mutant p53 phenotype was defined by the absence of staining (null; threshold of 5%) or > 80% of positive cells in the tumor.^23,24^ Assignment of p16 status (positive vs negative) was defined using the most recently published guidelines from Lewis Jr. *et al.* ^25^ of > 70% strong nuclear and cytoplasmic staining as identified by the primary pathologist involved in the individual cases. High risk HPV RNA in situ hybridization was performed using the HPV-HR18 cocktail (Advanced Cell Diagnostics, Newark, CA) using standard protocols for 2.5 HD Assay-Brown. This assay hybridizes to the E6/E7 mRNa for the following high risk HPV types: 16,18,26,31,33,35,39,45,51,52,53,56,58,59,66,68,73 and 82. HPV16 mRNA positive OPSCC was used as a control. DAB positivity of the HPV-HR18 probe in any part of the tumor area were scored as positive for HPV.

### Computerized analysis of tissue specimens.

Hematoxylin and eosin (H&E) stained slides were digitized by scanning using Ventana iScan HT at 40x resolution (0.25 um/pixel resolution). HistoQC^26^, an open-source quality control tool for digital pathology slides, was used to identify cases inappropriate for the study due to the presence of large blurry areas, obstructive dotting pen markings, or subcoverslip bubbles. Multinucleation frequency was calculated using a previously described algorithm^17^, which generated a multinucleation index (MuNI) characterizing the density of multinucleation events in epithelial regions. Tumor infiltrating lymphocyte (TIL) frequency and intra-tumoral distribution was quantified using a validated machine learning algorithm (OP-TIL).^18^ The MuNI and OP-TIL risk scores were computed as continuous variables for each patient and a cutoff was then used to stratify patients into low- and high-risk categories as described in previous works.^17,18^

## Results

### Patient and disease characteristics.

We reviewed data from a total of 56 male patients with a diagnosis of OPSCC, with a mean age of 59.5, a heavy tobacco exposure history (Table 1; median pack year history = 40), and a high rate of nodal metastasis (45/56, 80%) at time of presentation. Approximately 50% (28/56) of patients experienced local recurrence exclusively, 15 developed regional recurrence exclusively, and 1 patient developed a combination of local and regional recurrence. Distant metastasis occurred in 7 patients, of which 6 occurred in the lung; 7 patients developed both distant metastasis and locoregional recurrence concomitantly. Mean time to locoregional recurrence was 1.52 years (range 0.35–11.48 years); mean time to distant metastasis was 0.70 years and to combined recurrence 2.26 years.

p16 and p53 concordance between primary and recurrent/metastatic disease. Quantitative p16 immunohistochemistry was used to categorize 41 tumor pairs as p16 positive (+) or p16 negative (−) based on the current clinical standard of > 70% strong nuclear and cytoplasmic staining^25^: 14/41 (34%) primary tumors and 10/41 (24%) recurrent tumors were p16+. Eight tumors maintained p16 + status at time of recurrence. Twenty-three tumors maintained p16-negative status at time of recurrence. Six tumors converted from p16 + to p16− and 2 tumors converted from p16− (detectable but < 70%) to p16+ (> 90%). Among the tumors which converted from p16 + to p16−, one was a primary site recurrence 8 years post initial treatment and 5 were nodal recurrences which occurred < 24months following treatment completion. One primary tumor converted from p16− to p16 + 7 years later and one p16-primary generated a p16 + lung metastasis < 1 year following treatment. Overall, we detected a very high rate of p16 concordance between primary and recurrent disease. To validate the quantitative p16 IHC data we analyzed the tumors for high-risk HPV mRNA. Of the 41 primary tumors with both p16 and HPV data, concordance was 91%; of the 43 recurrent tumors with both p16 and HPV data, concordance was 95%. All 6 discordant tumors among the 84 analyzed had p16 staining > 70% but negative HPV staining.

Although *TP53* sequencing is the gold standard for determining mutational status for this critical tumor suppressor, p53 IHC has demonstrated high concordance with mutational analysis across multiple tumor sites and patient cohorts.^27–29^ Forty tumor pairs were analyzed in parallel for p53 and p16 expression levels. We identified 24 primary tumors as likely *TP53* mutant based on staining patterns along with 22 recurrent tumors; there were 15 concordant mutant-mutant pairs and 9 concordant wildtype-wildtype pairs. Among tumors with > 80% p53 positive cells, none demonstrated positive p16 status; the concordance decreased for tumors with < 5% p53 positive cells, with 8 tumors demonstrating both < 5% p53 positive cells and p16 positivity.

Multinucleation concordance between primary and recurrent/metastatic disease. Multinucleation scores (MuNI) were analyzed for 47 tumor pairs. Mean MuNI scores were 0.00032 for primary tumors (range 0-0.00083) with mean MuNI score for recurrent tumors of 0.00037 (0-0.00062). We previously defined two MuNI thresholds as a function of survival, one of 0.00038 based on a large, multi-institutional OPSCC patient cohort^17^ and a second threshold recently defined by our group at 0.00046 based on tumor biology (threshold defines > 90% of HPV-associated tumors).^30^ Using the 0.00046 cutoff 9 primary tumors were considered high-risk and 15 recurrences were considered high-risk. Across the time period, 19 tumors remained low-risk (40%), 14 converted from low-risk to high-risk (30%), 7 remained high-risk (15%) and 7 converted from high-risk to low-risk (15%). When limiting the analysis to primary site recurrences only (25), 10 tumors remained low-risk (40%), 7 converted to high-risk (28%), 4 remained high-risk (16%) and 4 (16%) converted to low-risk. Differences in MuNI between primary and recurrent tumors did not reach significance across all tumors (paired t-test 0.07), when limited to just primary site recurrences (0.084) or when limited to primary + regional recurrence (0.06). This was confirmed using chi-square analysis dichotomizing tumors as low-risk *vs* high-risk using either the 0.00046 or the 0.00038 thresholds.

Immune infiltration concordance between primary and recurrent/metastatic disease. We utilized 2 overlapping approaches to measure immune infiltration of paired tumors. First, we quantitatively assessed CD3 + and CD8 + TIL levels in the tumors as previously described.^8^ Mean CD3 + infiltration was 10.7% and 10.9% of primary and recurrent tumors (expressed as a % of total intra-tumoral nuclei) (paired t-test p = 0.96); mean CD8 + infiltration was 5.9% and 9.9% respectively (paired t-test p = 0.025). Mean PD-L1 positivity was 2.4% for primary tumors and 4.0% for recurrent tumors (paired t-test p = 0.04). Eight primary tumors exhibited ≥ 1% positivity and 21 recurrent tumors exhibited ≥ 1% positivity. Only 1 primary tumor converted from ≥ 1% to < 1% upon recurrence. All other tumors retained ≥ 1% positivity.

Second, we calculated OP-TIL scores for primary and recurrent tumors as previously outlined^18^ and dichotomized tumors into high-risk and low-risk. Chi-square analysis demonstrated no difference in OP-TIL scores between primary and recurrent tumors (chi-square statistic is 2.26; p-value = 0.13). When combining MuNI and OP-TIL scores for primary and recurrent tumors, chi-square analysis again demonstrated no significant difference between primary and recurrent tumors (chi-square statistic is 0.09; p-value = 0.77). OP-TIL status changed (high-to low-risk or vice versa) in only 18/46 pairs.

## Discussion

Current approaches to the treatment of head and neck cancer are predicated on the basic concept that treatment delivery, whether consisting of chemotherapy, radiation or chemo-radiation, alters the intrinsic biological background of the tumor along with the tumor immune microenvironment. For radiation-based treatment, repeated application of radiation is limited to use for treatment of patients whose tumors demonstrate recurrence after a prolonged disease-free interval^31,32^ in large part due to the high fraction of patients who either develop ≥ Grade 3 toxicity and or have limited survival benefit. In both oropharyngeal and laryngeal squamous cell carcinoma patients, surgical salvage efficacy is strongly correlated with disease-free interval following initial treatment completion.^33,34^ Disease-free interval is also considered in the context of re-application of cytotoxic chemotherapy combined with immune checkpoint inhibitors. In CheckMate 141, patients with recurrent disease were referred to as “platinum refractory”, and the study utilized tumor progression or recurrence within 6 months of last platinum administration as an inclusion criteria for nivolumab administration.^35^ Conversely, KEYNOTE-048 which tested pembrolizumab alone or in combination with cytotoxic chemotherapy compared to cytotoxic chemotherapy alone excluded patients which had progressive disease within 6 months of curatively intended systemic treatment for locoregionally advanced disease.^36^

Driving these clinical decisions is the assumption that a tumor, once exposed to chemo-radiation, that persists after treatment cessation will demonstrate molecular features of chemo-radioresistance, which would limit the expected utility of additional treatment. Our data sheds some light on whether delivery of curative intent chemo-radiation impacts tumor biology through two phenotypic features that we have previously robustly linked to oncologic outcomes, namely anti-tumor immunity and tumor cell multinucleation. Overall, tumors remained stable following treatment, without a upward drift in multinucleation nor a downward drift in TIL infiltration when assessed using quantitative immunohistochemistry for CD3 + and the cytotoxic CD8 + subsets. Recurrent tumors, in fact, demonstrated slightly higher levels of CD8 + TILs and higher levels of PD-L1 expression. Although CD8 + TIL and PD-L1 expression levels were higher in recurrent tumors, the OP-TIL algorithm, which combines TIL quantity with overall distribution and spatial organization within the tumor compartment, detected no significant differences between primary and recurrent tumors.

In contrast to our findings, So et al. ^37^ used paired tumor analysis combined with IHC and mRNA profiling to show that CD8 + TIL and PD-L1 expression levels decreased in a majority of recurrent head and neck squamous cell carcinoma (HNSCC) tumors compared to the primary tumors (although both parameters varied widely). A more focused approach was used by Pflumio et al. ^38^ to evaluate recurrence within a previously irradiated HNSCC field and showed lower CD3 + TIL levels and PD-L1 expressing cells in irradiated tumors but consistent CD8 + TIL levels. These discordant results explain in part the lack of consistent PD-L1-based CPS scoring trends between primary and recurrent tumors summarized by Girolami et al. ^39^ in a systematic analysis of the literature. Discordant datasets may in part be due to inclusion of different HNSCC sites (e.g., oral cavity, oropharynx, larynx) and variable HPV association.

Park et al. ^40^ recently used paired local recurrences for chemo-radiation treated HNSCC to show discordance of PD-L1 varied as a function of site with oral cavity demonstrating higher discordance and variable discordance depending on the CPS threshold chosen for analysis. Even when the selection for low levels of PD-L1 is maximal, as in the context of clinical trials utilizing anti-PD1/PD-L1 antibody-based strategies, concordance of anti-tumor immunity, whether measured via PD-L1 IHC or gene expression (e.g. interferon gamma, tertiary lymphoid structure signatures) is variable between primary and recurrent tumors.^41^

Intrinsic biological characteristics measured via p16 and p53 IHC appeared concordant between primary and recurrent tumors analyzed in the current study in contrast to data from several other studies (not limited to OPSCC) which showed reduction in mucin (MUC) 1 expression in recurrent tumors^42^ along with higher rates of p14(ARF) alterations (promoter methylation, point mutations)^43^, and a discordant *TP53* mutational pattern including shifts from wild-type to mutant status and loss of *TP53* mutation at time of disease recurrence.^44^ A very detailed analysis by Hedberg et al. ^45^ found an overall higher rate of primary and synchronous metastasis concordance using whole exome sequencing compared to primary tumors and their recurrent counterparts including enrichment of discoidin domain receptor tyrosine kinase 2 (*DDR2*) events.

Limitations of the current study include limited sample size and a limited scope of analysis as opposed to a more in depth transcriptomic and/or genomic analysis. However, in contrast to previous studies, ours is the first to limit the analysis to one HNSCC site, namely oropharynx and at the same time focus on patients who underwent curative intent radiation-based treatment. We focused our analysis on multinucleation and anti-tumor immunity because they represent previously validated phenotypic markers of treatment response and or effectiveness in the setting of primary OPSCC treated with either surgery or chemo-radiation.^8,16–18,30^ As a result, we expected that significant differences in these markers would provide more proximate phenotypic information about primary and recurrent disease. Future studies will expand both the number of paired samples and the depth of the analysis using spatial transcriptomic approaches that will allow us to not only better characterize potential changes in the tumor immune microenvironment but further unravel the relationship between multinucleation and infiltrating immunocytes. One limitation of the current study, shared by all other similar work stems from the nature of solid tumors themselves. Whereas in hematopoietic malignancies, interrogation of blasts during and immediately post-treatment makes it possible to rapidly assess development of resistant molecular backgrounds, in solid tumors, we are limited to sampling tumors prior to treatment and once the recurrent disease becomes clinically evident. Even when this process is temporally compressed, the number of population doublings between the end of treatment and development of measurable (~ 1cm) disease or persistence of viable tumor at first post-treatment scan (2–3 months post treatment completion) is massive. This extended duration raises the possibility that the impact of treatment on tumor biology may be diluted during subsequent tumor expansion. To overcome this intrinsic limitation, our ongoing studies couple conventional analysis outlined here with serial interrogation of cell free DNA and circulating exosome to continuously interrogate tumor biology during and following treatment.

## Conclusions

Data from the current study do not support a significant deleterious shift in either intrinsic tumor biology or anti-tumor immunity (assessed within the tumor volume) that could negatively impact the effectiveness of salvage therapeutic strategies.

## Figures and Tables

**Figure 1:**
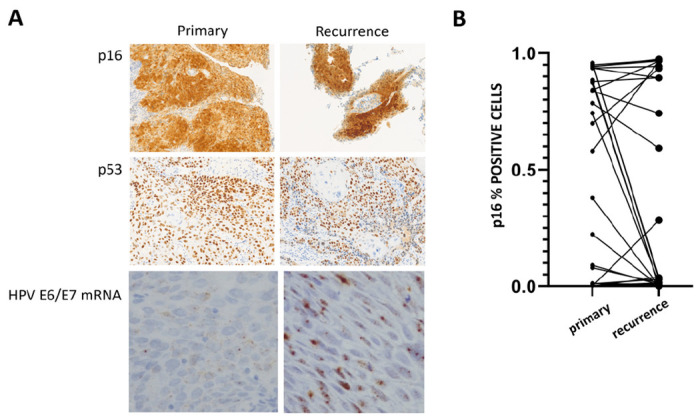
A) Representative staining of primary and recurrent tumors for p16, p53 and high risk HPV E6/E7 mRNA. B) Changes in p16 status between primary and recurrent tumors.

**Figure 2:**
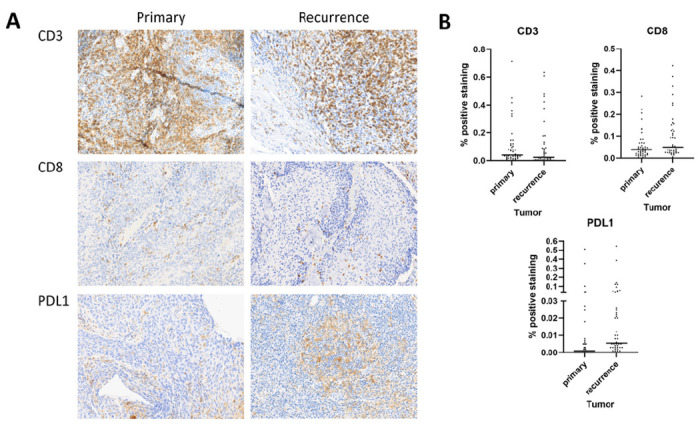
A) Representative staining of primary and recurrent tumors for CD3, CD8, and PDL1. B) Distribution of immune infiltration and PDL1 positive cells across primary and recurrent tumors.

**Table 1:** Patient and tumor characteristics.

Age	mean (years)	59.5
Gender	male	57
	female	0
Tobacco	yes	50
	no	7
Pack years	mean	41.4
	median	40
T-classification	1	10
	2	16
	3	17
	4	14
N-classification	0	11
	1	4
	2a	4
	2b	18
	2c	16
	3	3
